# Candidemia due to uncommon Candida species in children: new threat and impacts on outcomes

**DOI:** 10.1038/s41598-018-33662-x

**Published:** 2018-10-15

**Authors:** Ming-Horng Tsai, Jen-Fu Hsu, Lan-Yan Yang, Yu-Bin Pan, Mei-Yin Lai, Shih-Ming Chu, Hsuan-Rong Huang, Ming-Chou Chiang, Ren-Huei Fu, Jang-Jih Lu

**Affiliations:** 1 Division of Neonatology and Pediatric Hematology/Oncology, Department of Pediatrics, Chang Gung Memorial Hospital, Yunlin, Taiwan; 2 Division of Pediatric Neonatology, Department of Pediatrics, Chang Gung Memorial Hospital, Taoyuan, Taiwan; 3 Department of Laboratory Medicine, Chang Gung Memorial Hospital at Linkou, Taoyuan, Taiwan; 4 College of Medicine, Chang Gung University, Taoyuan, Taiwan; 5 Department of Medical Biotechnology and Laboratory Science, Chang Gung University, Taoyuan, Taiwan; 6 Biostatistics Unit of Clinical Trial Center, Chang Gung Memorial Hospital, Linkou, Taiwan

## Abstract

Many uncommon *Candida* spp. (species other than *C. albicans*, *C. parapsilosis*, *C. glabrata*, *C. tropicalis*, and *C. krusei*) have been shown to emerge in tertiary care facilities. We aimed to investigate these uncommon candidemia in children. Forty-six cases of candidemia caused by uncommon *Candida* spp. were identified during 2003–2015 from a medical center in Taiwan. The most common specie was *C. guilliermondii* (31.2%), followed by *C. lusitaniae* (18.8%) and *C. metapsilosis* (18.8%). These cases were analyzed and compared with 148 episodes of *C. albicans* candidemia. The incidence density of uncommon *Candida* spp. candidemia and the proportion to all candidemia episodes increased substantively during the study period. Prior exposure to azoles was uncommon in the 30 days prior to infection, but fluconazole resistant strains were significantly more common (n = 19, 41.3%). The increased incidence density of uncommon *Candida* spp. candidemia was associated with increasing use of antifungal agents. No differences in demographics, underlying comorbidities, risk factors, clinical features, dissemination, and 30-day mortality were found between uncommon *Candida* spp. and *C. albicans* candidemia. Patients with uncommon *Candida* spp. candidemia were more likely to require modifications in antifungal treatment and receive echinocandin drugs (43.5% vs 21.6%, p = 0.007). Candidemia caused by uncommon *Candida* spp. had poorer response to antifungal treatment, led to longer duration of candidemia (median 4.0 versus 2.5 days, p = 0.008), and had a higher treatment failure rate (56.5% vs 38.5%, p = 0.040).

## Introduction

Candidemia is a major cause of morbidity and mortality in the health care setting, especially among critically ill or immunocompromised patients or those with complicated medical conditions^1–3^. Among all *Candida*-associated invasive fungal diseases, *C. albicans*, *C. parapsilosis*, *C. tropicalis*, *C. glabrata*, and *C. krusei* account for nearly 90% of isolates from blood or sterile site cultures^1–6^. Candidemia caused by other uncommon species, including *C. haemulonii, C. guilliermondii*, and *C. lusitaniae*, is less well known and data have been reported only in small case series^7–12^. However, these uncommon fungal species have emerged as a new health threat to hospitalized patients and are endemic in some areas^10–12^. The widespread use of immunosuppressive therapies, broad-spectrum antibiotics, and antifungal prophylaxis may further increase the role of Candidal species as the causative pathogens among high-risk patients^13–15^.

Recently, clinical isolates of *C. haemulonii* and *C. guilliermondii* have been reported to exhibit decreased *in vitro* susceptibility to antifungal agents^9,16^, which highlights the importance of early identification and more updated treatment strategies. *C. lusitaniae* and *C. famata* have also presented as breakthrough candidemia in immunocompromised patients and lead to unfavorable outcomes^11,17,18^. Clinical data of invasive candidiasis caused by these uncommon yeasts have mostly come from adult patients^10–12,17,18^, whereas relevant studies reported in children are rare^9^. Because most institutions have had limited experience with candidemia caused by these uncommon *Candida* spp. in children, we conducted an observational study of all candidemia cases caused by these pathogens that occurred at our institution during a 13-year period.

## Materials and Methods

### Study design, collection of isolates and antifungal susceptibility

This study was part of a collaborative, combined retrospective and prospective collected database, laboratory-based, single-center study of invasive yeast infection as previously described^19^. We identified patients younger than 18 years of age with *Candida* bloodstream infection (BSI) caused by uncommon *Candida* spp. between January 2003 and December 2015. All *Candida* isolates were phenotypically identified by using the API 32C AUX yeast identification kit (bioMérieux SA, Marcy l’Étoile, France) and chromogenic culture media (CHROMagar, Becton Dickinson and Company, Franklin Lakes, NJ, USA). Beginning in December 2013, we applied Matrix-assisted laser desorption ionization time-of-flight mass spectrometry (MALDI-TOF, Bruker Biotype, software version 3.0, USA), ITS1-5.8S-ITS2 rDNA gene sequencing and large-subunit (18S) ribosomal RNA gene D1/D2 domain sequencing to re-confirm all these species. The study was approved by the Institutional Review Board and Human Research Ethics Committee of Chang Gung Memorial Hospital (CGMH), and a waiver of informed consent for anonymous data collection was also approved. All methods in this current study were performed in accordance with the relevant guidelines mentioned in this manuscript.

We also enrolled all cases of candidemia in children caused by *C. albicans* during the study period for comparisons. We excluded cases of unidentified *Candida* spp. and selected only the first isolate recovered from the blood if a patient had several cultures positive for the same *Candida* spp. Antifungal susceptibility of all these *Candida* isolates to nine antifungal agents was determined by broth microdilution method using a Sensititre YeastOne system (Trek Diagnostic Systems Ltd., East Grinstead, UK) according to the manufacturer’s instructions^20,21^. Minimum inhibitory concentration (MIC) was recorded as the highest concentration of antifungal agent resulting in the development of a blue color. The criteria for susceptibility of these *Candida* isolates to nine antifungal agents were based on MIC breakpoints of *Candida* spp. recommended by the Clinical & Laboratory Standards Institute (CLSI) guidelines^22^. For uncommon *Candida* spp., other than *C. guilliermondii*, clinical breakpoints are undefined; therefore, isolates that showed MICs higher than the epidemiologic cutoff value were considered potentially resistant^23^.

### Data collection and definitions

An incident episode of candidemia was defined as the first positive blood culture drawn from a peripheral vein yielding a *Candida* species, with clinical symptoms and signs compatible with Candida BSI^24,25^. Episodes were considered to be separate if they occurred at least 1 month apart and when at least one negative blood culture was noted between them. The clinical information was accumulated from a review of medical charts and included demographic characteristics, predisposing risk factors within the preceding 30 days from the onset of *Candida* BSI (defined as the day of first positive blood culture for *Candida* spp), underlying diseases, and the presence of an intravenous catheter or any other artificial device at the time candidemia appeared. The clinical manifestations at the time of blood culture collection, ICU admission, and the antimicrobial regimens used were also collected.

An episode of candidemia was considered catheter-related only if the same *Candida* species was cultured from the catheter tip during the episode; the definition was suggested by the guidelines of the Infectious Diseases Society of America^26,27^. Persistent candidemia was defined as repeated positive blood cultures for *Candida* spp. for more than 3 days after antifungal agents were administered. Candidemia-attributable mortality was defined as patients died within 7 days after onset of candidemia or in the presence of persistent clinical sepsis or persistent candidemia, or those died of candidemia associated complications^28,29^. Breakthrough candidemia was defined as new occurrence of candidemia while the patient was on antifungal agents for more than three days^30,31^. The primary study outcome was clinical treatment failure, which was defined according to the Mycoses Study Group and European Organization for Research and Treatment of Cancer consensus criteria^32^ as the following: (1) all-cause mortality between days 3 and 30 after the initial positive blood culture, or (2) persistent fungal BSI for ≥72 hours after the initiation of antifungal therapy. The secondary outcome was all cause in-hospital mortality. Patients’ response to antifungal therapy following candidemia was defined according to the consensus criteria of the Mycoses Study Group and European Organization for Research and Treatment of Cancer^33^.

### Statistical analysis

The demographic, clinical, outcome variables and the *in vitro* susceptibility data were summarized using the descriptive statistics. All statistical analyses were performed using IBM SPSS software (version 22.0; IBM SPSS Inc., New York, USA). Categorical variables were compared using the χ^2^ or Fisher’s exact test, and continuous variables by the Mann-Whitney *U* test. A P value of 0.05 was considered significant. Poisson regression and the Cochran-Armitage test were used for trend analysis of the annual BSI incidence densities and the proportions of candidemia caused by uncommon Candida spp., respectively. We also compared BSI incidence densities for three time periods; 2003–2006, 2007–2011, and 2012–2015, using Poisson distribution and test-based methods. The correlation between the annual use of antifungals and time was evaluated by using the Spearman correlation. The associations between the incidence densities of uncommon Candida spp. BSI and the annual use of antifungals (defined as daily doses per 1,000 patient-days) were evaluated by using Poisson regression.

We used Cox regression analysis to identify factors that were significantly associated with death. Clinically relevant parameters in the univariate analysis (P < 0.1) were included at multivariate regression analysis. The full model was reduced to a final model by using a stepwise elimination procedure. The proportional hazards assumption was tested graphically and by building time-dependent variables.

## Results

We identified 323 episodes of candidemia that occurred in hospitalized children over the 13-year study period. A total of 25 cultures that previously grew unspecified *Candida* spp. were rechecked by our ITS1-5.8S-ITS2 rDNA gene sequencing and MALDI-TOF and large-subunit (18S) ribosomal RNA gene D1/D2 domain sequencing. Twenty-one of the cultures were documented, and four unidentified *Candida* spp. were excluded. A total of 46 episodes of candidemia in 45 patients were caused by 10 uncommon *Candida* spp (Table 1). These data were compared with those reported for 148 episodes of candidemia caused by *C. albicans* in 136 patients.

**Table 1:** The uncommon *Candida* species causing 46 episodes of candidemia in children.

Pathogens	Total episode number, n (%)	Age category^&^		Years of occurrence			Treatment outcomes	
		Newborn	Children	2003–2006	2007–2011	2012–2015	Persistent candidemia*	Attributable mortality
*C. guilliermondii*	15 (31.2)	7	8	3	4	8	9 (60.0)	3 (20.0)
*C. lusitaniae*	7 (18.8)	2	5	2	3	2	4 (57.1)	1 (14.3)
*C. metapsilosis*	9 (18.8)	3	6	0	2	7	6 (66.7)	3 (33.3)
*C. orthopsilosis*	3 (6.3)	1	2	0	0	3	3 (100)	1 (33.3)
*C. haemulonii*	4 (8.3)	1	3	1	1	2	2 (50.0)	1 (25.0)
*C. lipolytica*	2 (4.2)	1	1	0	2	0	1 (50.0)	0 (0)
*C. dubliniensis*	2 (4.2)	0	2	1	0	1	1 (50.0)	0 (0)
*C. pelliculosa*	2 (4.2)	1	1	0	0	2	1 (50.0)	1 (50.0)
*C. duobushaemulonii*	1 (2.1)	0	1	0	1	0	1 (100)	1 (100)
*C. famata*	1 (2.1)	0	1	0	0	1	0 (0)	0 (0)
Total	46 (100)	16 (34.8)	30 (65.2)	7 (15.2)	13 (28.3)	26 (56.5)	28 (60.9)	11 (23.9)
Controls: *C. albicans*	148 (100)	50 (33.8)	98 (66.2)	58 (39.2)	53 (35.8)	37 (25.0)	58 (39.2)	35 (23.6)

^&^Newborn: from neonatal intensive care unit, age <3 months old; children: ward or pediatric intensive care unit, age 3 months-18 years old.

*Defined as repeated positive blood cultures for *Candida* spp. for more than 3 days after antifungal agents.

**Figure 1:**
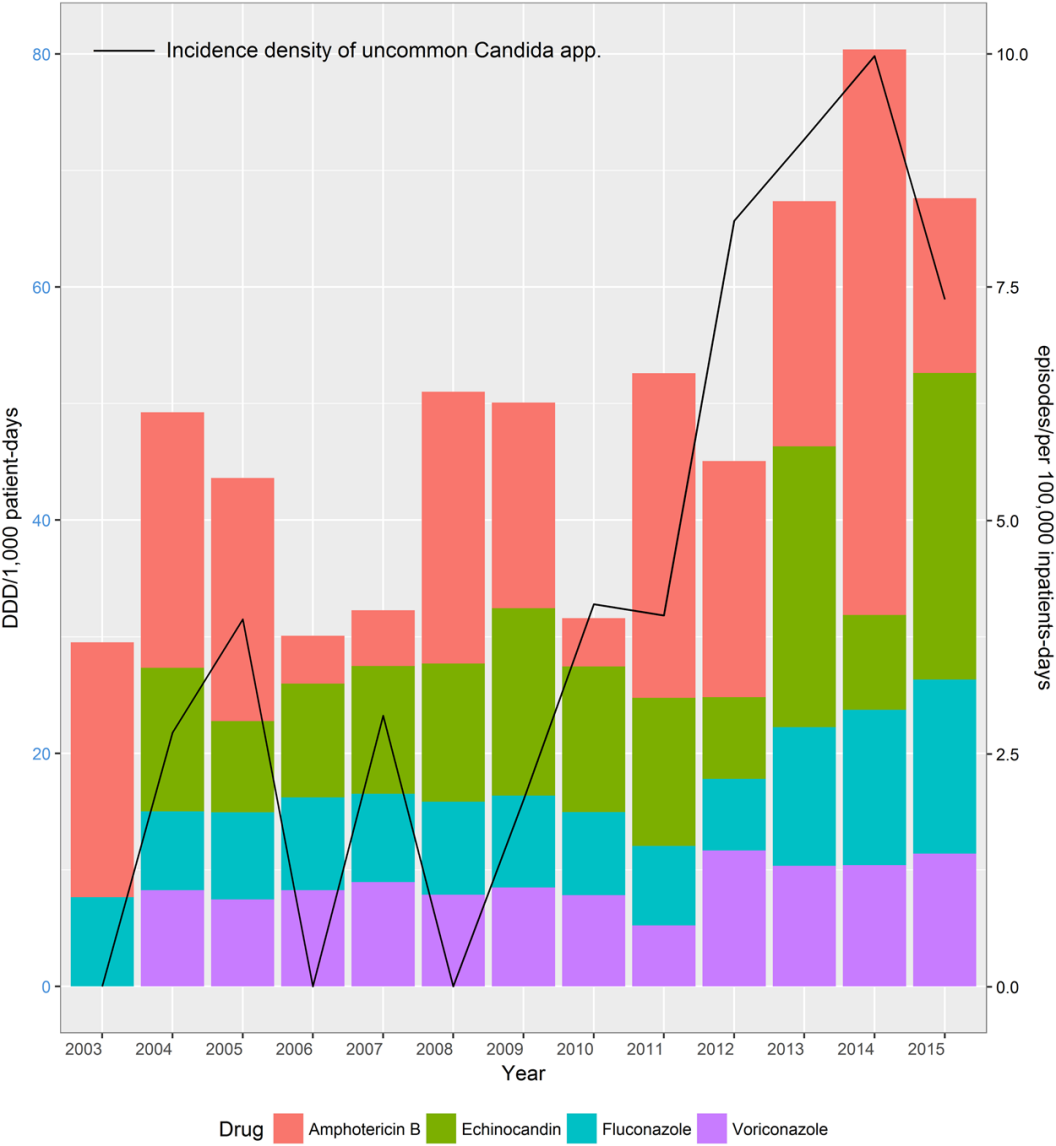
Increasing incidence density and proportion relative to all episodes of invasive candidiasis caused by uncommon *Candida* species and increasing annual use of overall antifungal drugs in Chang Gung Memorial Hospital, Taiwan, R.O.C. January 2003–December 2015. Spearman correlation coefficient r = 0.59, p = 0.033. DDD, defined dai.

The overall incidence of uncommon *Candida* spp. candidemia and their proportion relative to all episodes of candidemia increased significantly during 2003–2015 (incidence density p < 0.001; proportion p < 0.001) (Fig. 1). The overall incidence density of uncommon *Candida* spp. BSI was 3.61 episodes per 100,000 inpatient days, which increased from 1.48 (2003–2006) to 2.47 (2007–2011) and then to 7.29 (2012–2015; p < 0.001). Twenty-nine (63.4%) of the 46 episodes of uncommon *Candida* spp. candidemia occurred after January 2012. The overall proportion of uncommon *Candida* spp. candidemia relative to all episodes of candidemia in children was 14.4% and increased from 6.2% (2003–2006) to 9.3% (2007–2011) and then to 29.1% (2012–2015; p < 0.001). During 2012–2015, *C. guilliermondii* and *C. metapsilosis* had the highest incidence density (both 1.76 episodes/100,000 inpatient days) and had increased significantly compared with cases during 2003–2006 and 2007–2011. The incidence density rate of other uncommon *Candida* spp. did not increase.

Echinocandins have been available in our institute since 2004. Although the annual use of echinocandins, azole antifungals, and amphotericin B increased gradually during 2003–2015, there was no statistically significant increase in their use. The overall use of antifungal agents did increase significantly during this study period (Spearman r = 0.68; p = 0.040) (Fig. 1). The increase in incidence density of uncommon *Candida* spp. candidemia was correlated with the trend of increased voriconazole (VFEND®, Pfizer, New York, NY, USA) use (p = 0.098) and was significantly associated with the continuous increase in overall antifungal agent use (p = 0.033).

Most chronic comorbid conditions and associated risk factors were comparable between cases of candidemia caused by uncommon *Candida* spp. and *C. albicans* groups (Table 2). Although uncommon *Candida* spp. had a significantly higher MIC to fluconazole and relatively more commonly presented as breakthrough candidemia (17.4% vs 8.1%, P = 0.094), previous exposure to azoles was comparable between these two groups. The clinical characteristics, therapeutic regimens and treatment responses of uncommon *Candida* spp. candidemia and *C. albicans* candidemia are compared in Table 3. The severity of illness, judged by rates of severe sepsis and septic shock, were comparable between cases of candidemia caused by uncommon *Candida* spp. and those due to *C. albicans*. However, candidemia caused by uncommon *Candida* spp. led to significantly higher rates of persistent candidemia compared to *C. albicans* candidemia (76.1% vs 56.8%, p = 0.024).

**Table 2:** Demographic and clinical characteristics of 46 episodes of candidemia caused by uncommon *Candida* spp. versus 148 episodes of *C. albicans* candidemia.

Characteristic	Uncommon *Candida* spp. candidemia (total n = 46)	*C. albicans* candidemia (total n = 148)	P value
Neonatal episodes, n (%)	16 (34.8)	50 (33.8)	0.809
Patient age (days) of neonatal episodes, median (IQR)	28.5 (18.5–65.8)	25.5 (11.8–58.5)	0.213
Non-neonatal episodes, n (%)	30 (65.2)	98 (66.2)	0.809
Patient age (years) of non-neonatal episodes, years (IQR)	3.9 (1.1–11.1)	4.2 (1.0–8.2)	0.711
Sex, male subjects/female subjects	21 (45.7)/25 (54.3)	71 (48.0)/77 (52.0)	0.866
Underlying conditions*			
Congenital or genetic anomalies	5 (10.9)	15 (10.1)	0.886
Neurological sequelae	18 (39.1)	48 (32.4)	0.476
Cardiovascular disease	5 (10.9)	17 (11.5)	0.908
Chronic lung disease and/or pulmonary hypertension	16 (34.8)	48 (32.4)	0.858
Gastrointestinal sequelae	11 (23.9)	44 (29.7)	0.575
Renal sufficiency with/without dialysis	8 (17.4)	19 (12.8)	0.467
Hematological/Oncology cancer	9 (19.6)	23 (15.5)	0.503
Immunodeficiency	2 (4.3)	2 (1.4)	0.212
Autoimmune disease	1 (2.2)	5 (3.4)	0.680
Hepatic failure or cholestasis	0 (0)	6 (4.1)	0.165
Days of hospitalization before candidemia onset, median (IQR)	29.0 (14.8–50.0)	29.0 (14.3–55.5)	0.787
Sequences of episodes			0.608
First episode	39 (84.8)	131 (88.5)	
Recurrent episode	7 (15.2)	17 (11.5)	
Associated risk factors			
Receipt of systemic antibiotics^&^	44 (95.7)	138 (93.2)	0.735
Prior bacteremia^&^	27 (58.7)	67 (45.3)	0.130
Prior azoles exposure^&^	6 (13.0)	10 (6.8)	0.176
Presence of central venous catheter	45 (97.8)	141 (95.3)	0.683
Stay in an intensive care unit	31 (67.4)	110 (74.3)	0.549
Receipt of parenteral nutrition	30 (65.2)	94 (63.5)	0.863
Receipt of immunosuppressive drugs	14 (30.4)	29 (19.6)	0.154
Presence of artificial device other than central venous catheter	27 (58.7)	68 (46.0)	0.176
Prior surgery^&^	16 (34.8)	46 (31.1)	0.719
Neutropenia^¶^	14 (30.4)	31 (20.9)	0.230

All data were expressed as number (percentage %), unless indicated otherwise; IQR: interquartile range.

*Indicated the presence of underlying condition or risk factor at onset of candidemia, and most patients with candidemia had >1 underlying condition and/or risk factor.

^&^Within one month prior onset of candidemia, prior azoles exposure indicated patients received azoles drug in addition to the antifungal agents at time of candidemia.

^¶^Absolute neutrophil count ≤ 500 cells/*μ*L.

**Table 3:** Clinical features, treatment and outcomes of candidemia caused by uncommon *Candida* spp. versus *C. albicans* candidemia.

	Uncommon *Candida* spp. candidemia (total n = 46)	*C. albicans* candidemia (total n = 148)	P value
Clinical features			
Severe sepsis	18 (39.1)	55 (37.2)	0.862
Septic shock	15 (32.6)	44 (29.7)	0.717
Progressive and deteriorated candidiasis^¶^	6 (13.0)	33 (22.3)	0.209
Disseminated candidiasis^#^	0 (0)	7 (4.7)	0.133
Breakthrough candidemia	8 (17.4)	12 (8.1)	0.094
Duration of candidemia (days), median (interquartile range)	4.0 (1.8–8.3)	2.5 (1.0–5.0)	0.008
≤2 days	16 (34.8)	74 (50.0)	
3–7 days	17 (37.0)	52 (35.1)	
≥8 days	13 (28.3)	22 (14.9)	
Ultimate antifungal regimens for treatment			0.054
Fluconazole/Voriconazole	16 (34.8)	63 (42.6)	
Amphotericin B	9 (19.6)	44 (29.7)	
Echinocandins	20 (43.5)	32 (21.6)	0.007
Combination antifungal treatment	0 (0)	2 (1.4)	
None	1 (2.2)	7 (4.7)	
Effective antifungal agents given within 48 hours after onset of candidemia (based on antifungal susceptibility testing)	16/46 (34.8)	58/144 (40.3)	0.601
Total treatment duration (days), mean (interquartile range)	20.0 (14.0–27.5)	16.0 (14.0–22.0)	0.116
Catheter removal	23/45 (51.1)	91/141 (64.5)	0.107
Removal of central venous catheter within 3 days of onset	16/45 (35.6)	60/141 (42.6)	0.406
Treatment outcomes			
Responsiveness after initiation of antifungal treatment*			0.007
Within 72 hours	8 (17.4)	72 (48.6)	
4–7 days	15 (32.6)	22 (14.9)	
More than 7 days	10 (21.7)	20 (13.5)	
Treatment failure	26 (56.5)	54 (36.5)	0.040
Modification of antifungal treatment	29/45 (64.4)	54 (36.5)	0.003
Duration of candidemia after effective antifungal agents (days), median (IQR)	3.0 (1.0–7.5)	1.0 (1.0–4.0)	<0.001
Candidemia attributable mortality	11 (23.9)	35 (19.0)	0.971
Early mortality (≤7 days)	6 (13.0)	17 (11.5)	0.941
Late mortality (8–30 days)	5 (10.9)	18 (12.2)	0.929
Final in-hospital mortality	19/45 (42.2)	52/136 (38.2)	0.486

All data were expressed as number (percentage %), unless indicated otherwise.

^¶^Defined as candidemia episodes with more disseminated candidiasis and/or progressive multi-organ failure even after effective antifungal agents.

^#^Indicated positive *Candida* isolates recovered from more than two sterile sites, in addition to primary bloodstream infection.

*Responsiveness to antifungal agents was defined according to the consensus criteria of the Mycoses Study Group and European Organization for Research and Treatment of Cancer^33^.

Overall, 186 episodes (95.9%) of candidemia were treated with specific antifungal agents. The mean duration between onset of candidemia (time of the first positive blood culture for *Candida* spp.) and initiation of antifungal agents was 2.3 days (range 0–8 days). In 83 episodes (44.6%) of the 186 episodes, antifungal regimens were modified during the treatment course. Patients with uncommon *Candida* spp. candidemia had a significantly higher rate of antifungal regimens modification than did those with *C. albicans* candidemia, and were more often treated with echinocandins (43.5% vs 21.6%, p = 0.007). After modification of antifungal regimens, uncommon *Candida* spp. led to a significantly longer duration of candidemia (median 3.0 versus 1.0 days, p < 0.001), were slower to respond to antifungal agents, and had significantly higher rates of clinical treatment failure (56.5% versus 38.5%, p = 0.040), although the deaths due to fungemia and final in-hospital mortality were comparable between these two groups.

MICs for uncommon *Candida* spp. of eight antifungal agents are shown in Table 4. Overall, 19 (47.5%) of 40 available uncommon *Candida* spp. isolates were intermediate or resistant (minimum inhibitory concentration [MIC] ≥ 4 mg/L). With the exception of three C. *haemulonii* isolates that had high MICs of amphotericin B (2.0 mg/L), all other strains were susceptible to amphotericin B (MIC ≤ 1.0 mg/L). Seven *C. guilliermondii* isolates, three *C. haemulonii* isolates, and one isolate each of C. *lusitaniae*, *C. metapsilosis*, *C. orthopsilosis*, *C. pelliculosa* and *C. duobushaemulonii* were resistant (MIC ≥ 1 mg/L) or susceptible-dose-dependent (MIC 0.25–0.5 mg/L) to itraconzole. Except for 2 *C. haemulonii* strains, all isolates were susceptible to voriconazole (MIC ≤ 1 mg/L) and demonstrated posaconazole MICs of ≤0.5 mg/L. Caspofungin and micafungin demonstrated good activity against all species and isolates; an exception to this was one episode of *C. guilliermondii* with MICs of 8 mg/L and 2.0 m/L, respectively.

**Table 4:** Available susceptibility data for uncommon Candida isolates associated with candidemia in children.

Agents	MIC (mg/L)	*Candida guilliermondii*	*Candida lusitaniae*	*Candida metapsilosis*	*Candida orthopsilosis*	*Candida haemulonii*	*Candida lipolytica*	*Candida dubliniensis*	*Candida pelliculosa*	*C.duobushaemulonii*
		(n = 15)	(n = 7)	(n = 8)	(n = 3)	(n = 3)	(n = 1)	(n = 2)	(n = 2)	(n = 1)
AMB	MIC_50_	0.25	0.25	0.5	0.5	2.0	0.25	0.5	0.25	1
	MIC_90_	0.25	0.5	1	1	2.0	0.25	0.5	0.25	1
	Range	≤0.12–0.5	≤0.12–0.5	0.5–1	0.5–1.0	2.0	0.25	0.5	0.25	1
FLU	MIC_50_	4	1	2	2	>128	2	1	4	32
	MIC_90_	8	1	8	2	>128	2	1	4	32
	Range	2–8	0.5–16	1–8	1–2	16–>128	2	1	4	32
ITC	MIC_50_	0.25	0.06	0.12	0.12	>16	0.12	0.06	0.25	0.5
	MIC_90_	0.5	0.25	0.25	0.25	>16	0.12	0.06	0.25	0.5
	Range	0.12–0.5	0.03–0.25	0.06–0.25	0.12–0.25	1–>16	0.12	0.06	0.25	0.5
VOR	MIC_50_	0.06	0.015	0.06	0.06	>8	0.06	0.015	0.12	0.25
	MIC_90_	0.12	0.12	0.25	0.12	>8	0.06	0.015	0.25	0.25
	Range	0.03–0.25	≤0.008–0.12	0.03–0.25	0.06–0.25	0.5–>8	0.06	0.015	0.12–0.25	0.25
POS	MIC_50_	0.25	0.03	0.06	0.12	>8	0.25	0.03	0.5	0.5
	MIC_90_	0.25	0.06	0.12	0.12	>8	0.25	0.03	0.5	0.5
	Range	0.06–0.5	0.015–0.06	0.015–0.12	0.06–0.12	0.5–>8	0.25	0.03	0.5	0.5
5-FC	MIC_50_	≤0.06	≤0.06	≤0.06	0.12	<=0.06	0.5	<=0.06	<=0.06	>64
	MIC_90_	≤0.06	≤0.06	0.12	0.25	<=0.06	0.5	<=0.06	<=0.06	>64
	Range	≤0.06	≤0.06	≤0.06–0.12	≤0.06–0.25	<=0.06	0.5	<=0.06	<=0.06	>64
CAS	MIC_50_	0.25	0.25	0.25	0.5	0.12	0.06	0.12	0.03	0.06
	MIC_90_	0.5	0.5	0.25	0.5	0.25	0.06	0.12	0.06	0.06
	Range	0.06–>8	0.12–0.5	0.12–0.25	0.5	0.06–0.25	0.06	0.12	0.03–0.06	0.06
MIC	MIC_50_	0.5	0.06	0.5	0.5	0.25	0.25	0.015	0.015	0.12
	MIC_90_	1	0.06	0.5	1	0.25	0.25	0.015	0.03	0.12
	Range	0.25–2	0.03–0.06	0.12–0.5	0.25–1	0.06–0.25	0.25	0.015	0.015–0.03	0.12

AMB: amphotericin B; CAS: caspofungin; FLU: fluconazole; 5-FC: 5-flucytosine; ITC: itraconazole; MIC: micafungin; POS: posaconazole; VOR: voriconazole.

## Discussion

Population-based surveillance studies have documented the shift of candidemia from *C. albicans* to non-*albicans* species over the past two decades^2,4,5^. Uncommon *Candida* species, which generally account for less than 10% of all cases of candidemia^17,18^, are emerging among critically ill patients. The reported prevalence of uncommon *Candida* spp. in children varies widely between 3.2–22%, depending on definitions, geographic region, and patient characteristics^34–37^. We found that uncommon *Candida* spp. accounted for 14.4% of all cases of candidemia in children, and our result is in agreement with a recent study that documented the incidence and proportion of uncommon *Candida* spp. BSIs has risen during the past decade. We found uncommon *Candida* spp. candidemia were more frequently associated with treatment failure than candidemia caused by *C. albicans*, as these isolates were more commonly resistant to azoles, which led to poorer response and longer duration of candidemia.

In contrast to non-*albicans* candidemia in adults, in which prior fluconazole exposure was often concluded as an independent risk factor^18,38,39^, studies in the pediatric populations found no difference between *C. albicans* and non-*albicans* candidemia in terms of demographics, underlying disease, risk factors, clinical features and outcomes^32,40^. However, previous studies attempting to investigate the risk factors of non-*albicans* candidemia in children have focused on the more common *Candida* spp., such as *C. parapsilosis* and *C. glabrata*^32,41,42^. To our knowledge, this is the first study to investigate uncommon *Candida* spp. candidemia in children. We found similar patients characteristics, risk factors and comparable outcomes between uncommon *Candida* spp. and *C. albicans*. These results are in agreement with other reports concluding host characteristics and underlying medical illness are the most powerful predictors of final outcomes^14,42–44^.

The overall incidence of candidemia during the study period remained stable and was not affected by the changes in antifungal treatment policies^45,46^. It is tempting to speculate that an increase in strains resistant to fluconazole and higher MICs values were associated with the changes of in antifungal treatment policies. Published guidelines encourage empiric use of echinocandins in patients with severe illness, history of azole exposure, or neutropenia^47^. This may have influenced the anti-fungal prescribing practices. It is possible that a significant increase in consumption of antifungal agents during the first half of the study period accounted for the emergence of uncommon *Candida* species, which required longer periods of antifungal treatment and led to a vicious cycle of more uncommon *Candida* species. Therefore, the incidence density of uncommon *Candida* spp. BSIs was noted to be significantly higher after 2012.

During the 13-year study period, the approach and antifungal treatment policies at our institute changed in two aspects. In the neonatal intensive care unit, antifungal prophylaxis with fluconazole for extremely low birth weight infants was launched in 2011, and echinocandins became available since 2004. Caspofungin (Cancidas®, Merck, Sharp & Dohme, Kenilworth, NJ, USA) has been widely used since 2005 and micafungin (Micamine®, Astellas Pharma, Inc., Tokyo, Japan) became more common beginning in 2009. These changes in antifungal regimens may account for the changing epidemiologic characteristics of candidemia in children since 2011. Several studies have concluded that the increase of certain non-*albicans* or uncommon *Candida* species, such as *C. glabrata* and *C. kefyr*, are associated with the increasing use of echinocandin drugs^17,42,45^. Another study found significant positive correlation between use of itraconzaole and the increased incidence of *C. parapsilosis* and *C. guilliermondii* candidemia^45^. However, no antifungal agent can account for the emergence of uncommon *Candida* spp. candidemia found in this study. In addition, our cases of uncommon *Candida* spp. candidemia were less commonly breakthrough candidemia^17^ or due to previous treatment with specific antifungal agents.

In several studies, *C. guilliermondii* has been the most commonly isolated uncommon *Candida* spp. among pediatric patients^16,24,48^, and *C. lusitaniae* was common in another international study in children^33^. In other recent reports, more than half of all patients with candidemia caused by uncommon *Candida* spp. had breakthrough infections or underlying hematological malignancies^15,17^. In our cohort, almost all cases of pediatric candidemia had specific chronic comorbidities and the majority of breakthrough candidemia cases were due to *C. parapsilosis* and *C. glabrata*. These differences are a further reflection of the changing epidemiologic characteristics of pediatric candidemia and unique features of uncommon *Candida* spp. in children. Therefore, we concluded that uncommon *Candida* spp. distributions and clinical characteristics vary by patient population, geographic region, and antifungal practices^15,17,49^.

Our study had some limitations. First, it was a retrospective study from a single institution with a small number of episodes caused by individual uncommon *Candida* spp.; therefore, further multicenter, prospective studies, or systemic review with meta-analysis are required to update information applicable to different geographic areas or specific groups at risk for candidemia caused by uncommon *Candida* species. Second, although we used the MALDI-TOF and DNA sequencing to re-identify all *Candida* isolates in the past 13 years, some *Candida* strains of pediatric candidemia more than five years ago were not available and were identified phenotypically at that time. Therefore, it is possible that during 2003–2011, some *C. dublinensis* and other *Candida* isolates were mis-identified as *C. albicans*, and the frequency of uncommon *Candida* spp was thus underestimated. Although MALDI-TOF has strengths of rapid, sensitive, and economical in terms of both costs and labor involved, it is also limited that the spectral database of MALDI-TOF must contain peptide mass fingerprints of the specific species before it can correctly identify new species^50^. Finally, the uncommon *Candida* species are a heterogeneous “mixture” of many different organisms. Therefore, they may not share common clinical characteristics and treatment strategies should depend on individual cases.

In conclusion, uncommon *Candida* spp. causing candidemia are emerging among hospitalized children. We did not find clinical variables that enable us early recognition or prediction of candidemia caused by these pathogens. Although clinical outcomes at day 30 are similar to those caused by *C. albicans*, uncommon *Candida* spp. more frequently result in prolonged fungemia and treatment failure. Because uncommon *Candida* species frequently show fluconazole MICs above the epidemiologic cutoff values, identification of all *Candida* organisms at the species level by advanced molecular methods is of value in guiding treatment directions.

## Conclusion

Uncommon *Candida* species have now emerged among hospitalized children. These pathogens frequently are not susceptible to fluconazole and had higher rate of treatment failure; echinocandins are the treatment choice.
